# Functional Protein Composition in Femoral Glands of Sand Lizards (*Lacerta agilis*)

**DOI:** 10.3390/molecules27072371

**Published:** 2022-04-06

**Authors:** Alejandro Ibáñez, Bozena Skupien-Rabian, Urszula Jankowska, Sylwia Kędracka-Krok, Bartłomiej Zając, Maciej Pabijan

**Affiliations:** 1 Department of Comparative Anatomy, Institute of Zoology and Biomedical Research, Faculty of Biology, Jagiellonian University, 30-387 Kraków, Poland; maciej.pabijan@uj.edu.pl; 2 Department of Ecology and Vertebrate Zoology, Faculty of Biology and Environmental Protection, University of Łódź, 90-237 Łódź, Poland; 3 Proteomics and Mass Spectrometry Core Facility, Malopolska Centre of Biotechnology, Jagiellonian University, 30-387 Kraków, Poland; bozena.skupien-rabian@uj.edu.pl (B.S.-R.); urszula.jankowska@uj.edu.pl (U.J.); 4 Department of Physical Biochemistry, Faculty of Biochemistry, Biophysics and Biotechnology, Jagiellonian University, 30-387 Kraków, Poland; sylwia.kedracka-krok@uj.edu.pl; 5 Institute of Environmental Sciences, Faculty of Biology, Jagiellonian University, 30-387 Kraków, Poland; bartlomiej.f.zajac@gmail.com

**Keywords:** proteomics, femoral glands, inter-individual variation, lipid metabolism, immune system, Lacertidae

## Abstract

Proteins are ubiquitous macromolecules that display a vast repertoire of chemical and enzymatic functions, making them suitable candidates for chemosignals, used in intraspecific communication. Proteins are present in the skin gland secretions of vertebrates but their identity, and especially, their functions, remain largely unknown. Many lizard species possess femoral glands, i.e., epidermal organs primarily involved in the production and secretion of chemosignals, playing a pivotal role in mate choice and intrasexual communication. The lipophilic fraction of femoral glands has been well studied in lizards. In contrast, proteins have been the focus of only a handful of investigations. Here, we identify and describe inter-individual expression patterns and the functionality of proteins present in femoral glands of male sand lizards (*Lacerta agilis*) by applying mass spectrometry-based proteomics. Our results show that the total number of proteins varied substantially among individuals. None of the identified femoral gland proteins could be directly linked to chemical communication in lizards, although this result hinges on protein annotation in databases in which squamate semiochemicals are poorly represented. In contrast to our expectations, the proteins consistently expressed across individuals were related to the immune system, antioxidant activity and lipid metabolism as their main functions, showing that proteins in reptilian epidermal glands may have other functions besides chemical communication. Interestingly, we found expression of the Major Histocompatibility Complex (MHC) among the multiple and diverse biological processes enriched in FGs, tentatively supporting a previous hypothesis that MHC was coopted for semiochemical function in sand lizards, specifically in mate recognition. Our study shows that mass spectrometry-based proteomics are a powerful tool for characterizing and deciphering the role of proteins secreted by skin glands in non-model vertebrates.

## 1. Introduction

Proteins are structurally complex macromolecules with a wide variety of functions. The chemical versatility of proteins makes them ideal candidates for intraspecific chemical communication, including their putative involvement in the social and sexual interactions of animals [1,2]. For instance, darcin—a male-specific protein—mediates female attraction towards male urine scent marks in mice [3]. In Urodela—e.g., salamanders and newts—proteinaceous pheromones are expressed in cutaneous and cloacal glands [4,5,6]. Indeed, many vertebrates possess integumentary glands that secrete a wide variety of chemical substances. Yet, the vast majority of molecules, especially proteins, remain uncharacterized for non-model vertebrates.

Lizards (order Squamata) have both an olfactory epithelium and Jacobson’s (vomeronasal) organ that discriminate a range of compounds and/or scents [7,8,9]. In addition, some species of lizards possess femoral glands (FGs), epidermal structures located on the ventral epidermis of the hind limbs that secrete a rich mixture of chemicals [10,11,12]. Femoral glands are usually dimorphic and, in general, more developed in males than in females [10,12,13]. Recent comparative analyses suggested that FGs and other epidermal glands may have played a role in the evolution of sociality in this vertebrate group [14]. Indeed, it is well known that FG secretions are involved in many social interactions (reviewed in [12]). For instance, they may be used to mark territories and establish dominance relationships in territorial species [15,16,17]. Additionally, accumulating evidence indicates that femoral secretions may provide information about the signaler’s condition, facilitating the assessment of potential partners and thus ultimately influencing mate choice [12,18,19].

In the desert iguana, *Dipsosaurus dorsalis*, up to four fifths of the compounds in FG secretions are proteins while just around one fifth are lipids [20]. Although the lipophilic fraction is less abundant than the proteinaceous one, the former has been characterized and relatively well studied in many lizard species [12,21,22]. Studies focusing on the proteinaceous fraction, especially concerning the role of proteins, are much more scarce [20,23,24,25,26,27]. Recent research has shown that femoral gland proteins may play an important role in individual recognition in lacertid lizards, and may therefore be involved in intraspecific communication [24,26]. In addition, the proteinaceous fraction of FGs has been studied in a few lizard species, showing species-specific protein patterns, giving further support to the idea that proteins may be involved in chemical signaling [27]. Nevertheless, FG secretions, and in particular proteins, might have other unknown functions as well. A recent study did not identify any proteins directly connected to chemical communication in the Galápagos marine iguana [28]. Unexpectedly, proteins from FG secretions were linked to the innate immune system response, indicating a potential defensive role against pathogenic agents or an inhibitory role towards microbial agents that could degrade chemosignals [28].

The sand lizard *Lacerta agilis* Linnaeus (1758) is widely distributed across Europe, inhabiting semi-open to open areas, such as dunes, heathland, meadows, farmland and forest margins, to heavily modified areas including roadsides, railway embankments, abandoned mineral extraction sites and vegetated urban areas [29]. This species often lives at high densities, with many lizards utilizing overlapping home ranges with numerous opportunities for encounters [29]. Confrontations may lead to visual displays and fights among individuals. The FGs of male sand lizards, as in many other lacertids [12], are most prominent during and immediately after the spring mating season. Matthey (1929) [13] demonstrated that FGs are sexual secondary organs regulated by testicular hormones that boost gland development in male sand lizards. Laboratory experiments showed that female sand lizards choose male odors based on their underlying MHC class I genetic background, aiding in communication among or within the sexes of this species [30]. Although major lipophilic classes of compounds are present in FG secretions of *L. agilis* [31], the protein has not been studied.

Here, we characterized the protein component of FG secretions in male sand lizards using a mass spectrometry-based proteomic approach. Our main goals were to identify proteins expressed in FG secretions and explore patterns of variation and stability of protein profiles among individuals. Then, we examined the potential functions of proteins identified in lizard FGs. As a general prediction, we expected a high diversity of proteins in FG secretions, including some that are involved in intraspecific communication. We deciphered the potential functions of proteins by using bioinformatics tools and compared our results with previous research on FG proteins.

## 2. Results and Discussion

### 2.1. Protein Identification and Inter-Individual Variation

**Figure 1:**
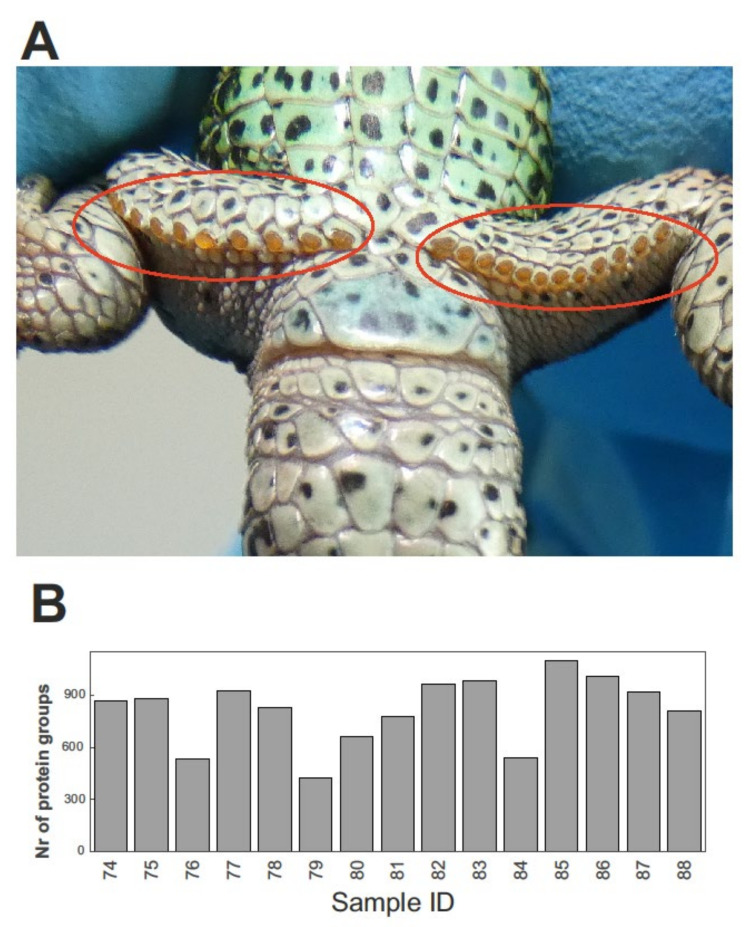
(**A**) Macroscopic aspect of femoral glands (highlighted with red circles) in a male sand lizard (*Lacerta agilis*). Photo by A. Ibáñez. (**B**) Total number of proteins identified in each femoral gland sample. Numbers in the abscissa are sample ID numbers (i.e., sample identifiers).

Femoral pores and yellow secretions produced by epidermal glands are easily discernible to the naked eye (see Figure 1A). Mass spectrometry-based proteomics allowed us to quantitatively profile the proteins present in the FG secretions of 15 male sand lizards. The average (±SD) number of identified protein groups—i.e., proteins grouped together according to their shared set or subset of peptides [32]—was 815 (±195) per FG sample. The variation among individuals was relatively high, ranging from 422 to 1097 identified protein groups (see Figure 1B).

**Figure 2:**
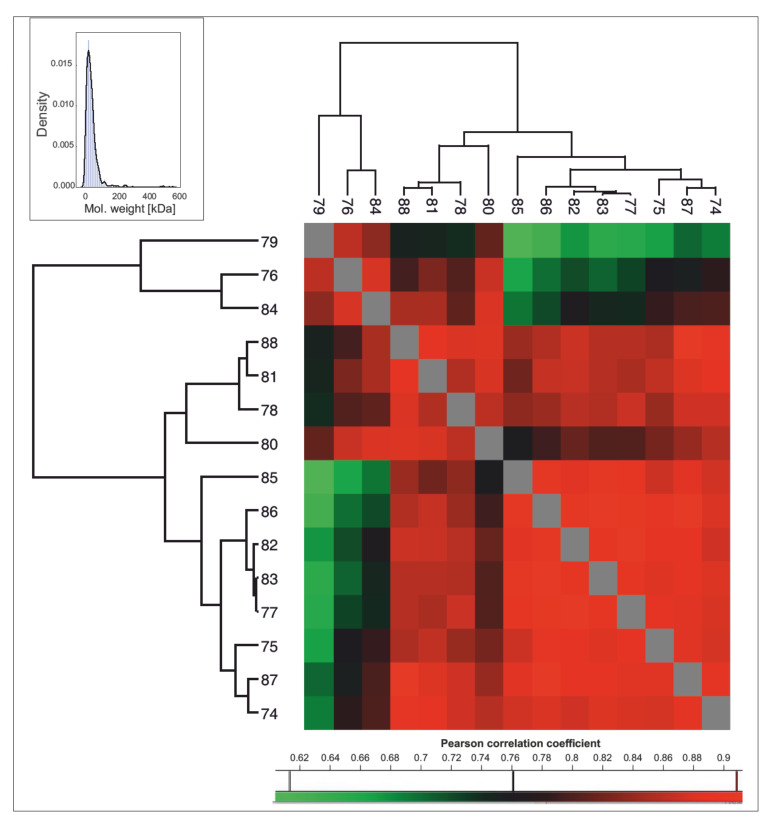
Top left: Density plot of the molecular weight (kDa) for protein groups with a valid iBAQ in at least 10 out of 15 samples (i.e., a total of 667 proteins selected). Heat map showing Pearson correlation coefficients for the 15 samples based on the iBAQ values of the 667 selected proteins. Scale shows color correspondence for the Pearson coefficient values.

Based on all identified proteins, there were 667 protein groups having valid (i.e., non “NaN”) intensity-Based Absolute Quantification (iBAQ [33]) values in at least 10 out of 15 samples (Supplementary File S1) that were selected for further inspection. These 667 protein groups have, in general, a relatively low molecular weight with a distribution similar to that of the marine iguana proteome (see Figure 2 and Tellkamp et al. [28] for a comparison). A relatively high correlation was observed across most of the samples according to iBAQ intensities for all 667 protein groups (Figure 2). These results show that, despite the variable number of proteins expressed in FGs, there is a subset of proteins more commonly expressed across individual lizards, indicative of functional stability (see Table 1 and below for proteins grouped by function). In the common wall lizard (*Podarcis muralis*), individuals from the same population or from the same color morphs possess similar electrophoretic banding patterns, probably due to genetic relatedness, but remarkable inter-individual variation in protein bands was also observed [24]. Although the large variability across samples in the total number of proteins in the sand lizard is in line with previous results (see Figure 1B), our findings also highlight the presence of a group of proteins that are more commonly expressed across individuals, whose functions are explored in more detail below.

### 2.2. Protein Functionality and Comparison with the Galápagos Marine Iguana Proteome

**Figure 3:**
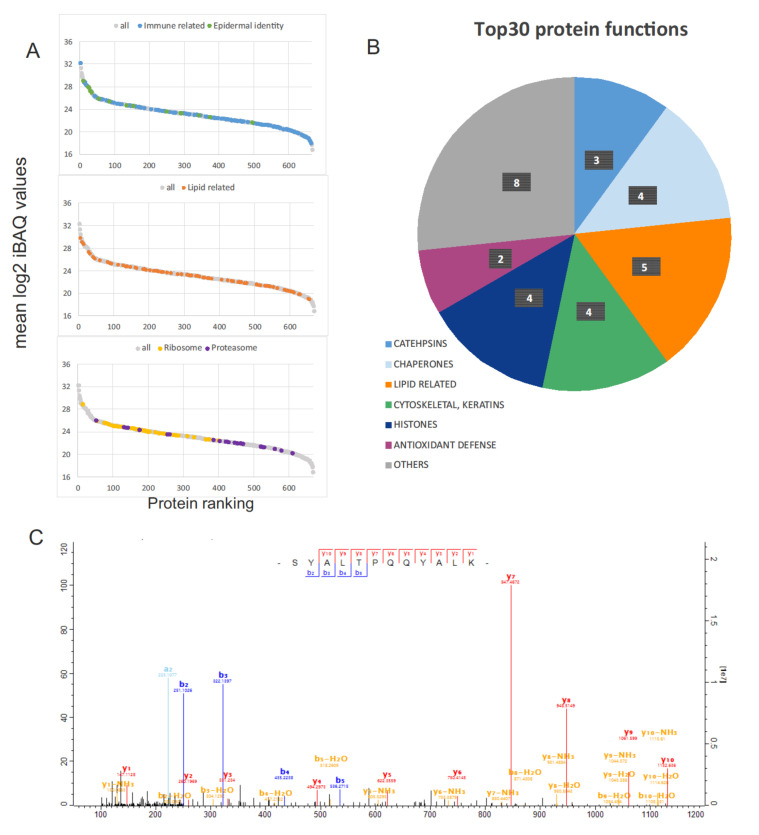
(**A**) Protein intensity rank (*x*-axis) based on the mean log2 iBAQ values (*y*-axis). Proteins of selected functional groups are color-coded (top: immune related and epidermal identity; middle: lipid related; bottom: ribosome and proteasome). (**B**) Detailed functions for Top30 proteins. The pie chart shows the number of proteins per category (see Table 1). (**C**) MS/MS spectrum of the peptide SYALTPQQYALK used for the identification of Cathepsin D (D3JHM6_9SAUR). The score value for this spectrum was 207.29.

The 667 protein groups were ranked according to the mean value of iBAQ intensity (Figure 3A). The 30 proteins with the highest iBAQ values (Top30) were grouped according to their functions in Table 1 (see Figure 3B for a visual representation of the proportion of Top30 proteins per functional category). We compared our results with the only other FG secretion proteome available, that of the distantly related Galápagos marine iguana [28].

The most abundant protein was cathepsin D (Figure 3C), which is a soluble lysosomal aspartic endopeptidase involved in numerous physiological processes, including degradation of intracellular proteins, activation and degradation of polypeptide hormones and growth factors, activation of enzymatic precursors, and brain antigen processing [35]. Another protein from the cathepsin family, cathepsin A, a serine protease [36], was also found among the Top30 proteins. Cathepsins play a significant role in immune responses through their ability to process antigens [37,38]. Cathepsin D was also one of the most abundant proteins found in FG secretions of the marine iguana [28].

The second most abundant protein in FG secretions of male sand lizards was carbonic anhydrase. This protein catalyzes conversion of carbon dioxide to bicarbonate, which might be responsible, e.g., for pH regulation [39].

Interestingly, the group of Top30 proteins contained two key antioxidant enzymes, superoxide dismutase (SOD1) and peroxiredoxin (PRDX4). Other antioxidant enzymes were repeatedly identified in our samples, including catalase with intensity rank (IR) of 112 and glutathione peroxidase (GPX6 and GPX1, IR of 180 and 252, respectively). These proteins may potentially have a protective role against the oxidation of the other components of FG secretion; e.g., antioxidants could protect lipids from peroxidation due to environmental agents. For instance, it has been hypothesized that differences in the lipophilic components of different populations of *Zootoca vivipara* could be driven by environmental humidity that may lead to the production of compounds preventing lipid oxidation in FG secretions [40]. Our results provide provisional evidence that proteins secreted in FGs may shield lipid-based semiochemicals from oxidative damage, and hence increase their resistance to environmental fade-out.

The Top30 proteins also included chaperones, cytoskeletal proteins, histones and others (see Table 1 and Figure 3B). As mentioned in Tellkamp et al. [28], the presence of intracellular proteins in FG secretion is expected due to the holocrine nature of the FGs. Moreover, proteins indicating the epidermal origin of FGs were detected in our experiment, as in Tellkamp et al. [28]. Several keratins (also in the Top30 group), proteins from the S100A family (IR: 23, 133 and 289), 14-3-3 sigma protein (IR: 84) as well as Tgm1 (IR: 373) were detected. Consistent with the hydrophobic nature of FG secretions, several lipid-related proteins were recognized as some of the most abundant (see Figure 3A,B).

The dataset of repeatedly identified proteins (667 protein groups) was also analyzed in terms of function using STRING (Search Tool for the Retrieval of Interacting Genes/Proteins; see Material and Methods). This analysis showed an enrichment in several biological processes, including immune system process (GO:0002376) and lipid metabolic process (GO:0006629) (see Supplementary File S1), which is in line with the study of the Galápagos marine iguana. KEGG (Kyoto Encyclopedia of Genes and Genomes) pathway enrichment also showed numerous ribosomal and proteasomal proteins in the dataset (see Supplementary File S1). Considering protein intensity within the samples, proteasomal proteins were less abundant than the other mentioned protein groups (Figure 3A). Immune- and lipid-related proteins were distributed across the whole range of intensity, while ribosomal and epidermal proteins attained higher positions in the intensity ranking (see Figure 3A). Analysis with the DAVID tool confirmed the STRING findings and revealed strong over-representation of extracellular exosome proteins within the analyzed FG protein set (proteins of the GO TERM “extracellular exosome” are shown in Supplementary File S2; see sheet “GO SLIMMER_ LIST OF TERMS”). Exosomes are membranous vesicles secreted by cells involved in intercellular signaling processes [41]. Femoral secretions are made up of components and organelles derived from cell degradation, with small vesicles also present [42,43]. Therefore, the observed over-representation of extracellular exosome proteins in FGs is a clear indication of secretory activity.

Our approach to the annotation of the sand lizard proteome relies on the use of high-quality but taxonomically restricted, annotated databases for human proteins. Our analysis therefore assumes similar gene functions in lacertids as in humans, which is a clear simplification. However, since only about 1% of protein sequences in the UniProt database have experimentally verified functions, the transfer of protein annotations between different species is a widespread approach [44,45,46,47]. Nonetheless, our results should be taken as a first approximation of the potential functions of FG proteins in sand lizards, and are in need of validation in future studies.

### 2.3. Are Sand Lizard FG Proteins Involved in Intraspecific Chemical Communication?

Contrary to our expectations, no proteins obviously linked to pheromones were found in this study. Several hypotheses could explain this result: (1) proteins related to chemical signaling remain in the non-identified proteinaceous fraction; (2) proteins might have multiple functions including a possible role in pheromone communication that cannot yet be discerned or annotated due to limitations in the databases; (3) proteins in sand lizard FGs are not directly involved in the semiochemical ecology of this species. Proteins related to lipid metabolism were well represented across the spectrum of functional groups (see Figure 3A). Among Top30 proteins, we found phospholipase A2 (EC 3.1.1.4) (IR: 24) and an aldo_ket_red domain-containing protein (IR: 28). These proteins might be connected to metabolism of the lipophilic compounds present in FGs. Phospholipases are enzymes that hydrolyze phospholipids into fatty acids and other metabolites. Aldo-keto reductase family members play a pivotal role in the biosynthesis and metabolism of steroids [48]. In the case of sand lizards, steroids and fatty acids account for roughly 50% of the lipophilic fraction of femoral secretions [31]. Therefore, phospholipases and aldo-keto reductases could be involved in the metabolism or production of specific lipids in FGs, e.g., steroids and fatty acids, that could be further used in chemical signaling [11,22]. In addition, a fatty acid binding protein (FABP5; IR: 41) was found among the 667 highly expressed proteins. Fatty acid binding proteins play an important role in the transport of some lipids. Although the connection of lipid metabolic proteins with chemical communication is unknown, these, or similar proteins, could be directly or indirectly involved in the biosynthesis and/or transport of chemosignals.

Antigen processing and presentation of exogenous peptide antigen via MHC (Major Histocompatibility Complex) class II (GO:0019886), as well as antigen processing and presentation of peptide antigen via MHC class I (GO:0002474), were detected among the multiple and diverse biological processes enriched in FGs (see Supplementary File S1). Previous research has demonstrated that the MHC class I genotype is correlated with mate acquisition and reproductive success in sand lizards [49]. Moreover, females perceive olfactory substances released by males, and prefer males with a more distinct MHC class I genotype [30]. The MHC genotype is associated with sexual secondary traits and chemical signaling in vertebrates [50]. Therefore, a tentative hypothesis is that MHC proteins present in FGs are recognized and used by females to choose potential partners (e.g., to choose males with a more distinct MHC background [30]). However, we note that caution should be used when interpreting GO enrichment analysis relying on human databases, for distantly related vertebrates. Whether MHC proteins might have a pheromonal function remains unknown, but our results provide a framework to further explore the potential connection between the FG proteome, MHC and mate choice.

To determine whether proteins are directly involved in intraspecific communication, large-scale studies on FG proteomics under a macroecological framework are needed. For instance, a good approach could be to first explore the relationship between chemical signal complexity (e.g., richness of proteins) and traits that are under sexual selection, such as body size, using a large number of lizard species [51]. In addition, other factors such as climatic or environmental conditions, as well as diet, could also influence protein composition in lizards. For example, in Schreiber’s green lizard (*Lacerta schreiberi*), it has been shown that experimental manipulation of the natural diet may affect the lipophilic composition of FG secretions, and could ultimately influence mate choice [52]. Similar experimental studies could be used to test whether diet may modulate protein expression in FGs. Additionally, further research integrating mass-spectrometry proteomics with evolutionary comparative analyses, as well as experimental studies, are necessary to shed light on protein functionality in the context of chemical signaling.

## 3. Materials and Methods

### 3.1. Lizard Capture and Sampling

Most lizards were collected within the city of Kraków in the surroundings of an old quarry (50.034° N, 19.865° E); only male sand lizards were sampled. Two animals were collected 30 km east of this area, at the edge of a forest (50.004° N, 20.274° E). Lizards were sampled in spring, coinciding with the mating season of this species. Femoral gland secretions were obtained by gently squeezing the femoral gland pores. The exudates were stored in 100 µL of lysis buffer (5% SDS, 0.1 M Tris, pH 7.5, 1 mM PMSF). The secretions were ground in tubes with a pestle, incubated at approximately 100 °C for 5 min and refrigerated before further processing. Proteome analysis was conducted in samples of femoral secretions originating from 15 male lizards.

The samples were sonicated for 15 min at 320 W (intensity setting—high) with time interval 30 s/30 s ON/OFF using a Bioruptor UCD-200 sonicator (Diagenode, Liege, Belgium). Then, samples were incubated at 95 °C for 5 min and centrifuged (20,000× *g*, 10 min, RT). Supernatants were saved. Protein digestion was performed using S-Trap™ micro spin columns according to the manufacturer’s protocol. Briefly, samples were solubilized in 5% SDS, 50 mM triethylammonium bicarbonate (TEAB; pH 7.55), reduced with 50 mM DTT solution, and alkylated with the addition of iodoacetamide to a final concentration of 40 mM, then acidified with phosphoric acid. Afterwards, 6 volumes of Strap binding buffer (90% aqueous methanol, 100 mM TEAB, pH 7.1) were added and the mixture was placed on the S-Trap by centrifugation at 4000× *g* for 10 s. Samples were purified by washing with S-Trap binding buffer and centrifugation. Proteins were digested overnight with trypsin (Promega, Madison, WI, USA) at 20:1 protein to enzyme wt:wt ratio. Peptides were eluted with 50 mM TEAB, followed by 0.2% aqueous formic acid and 50% acetonitrile, containing 0.2% formic acid. Lastly, peptides were vacuum dried.

### 3.2. LC-MS/MS Analysis

The LC-MS/MS analysis was conducted with the use of a nanoHPLC system (UltiMate 3000 RSLCnano System, Thermo Fisher Scientific, Waltham, MA, USA) coupled with a Q Exactive mass spectrometer (Thermo Fisher Scientific, Waltham, MA, USA). Peptides were suspended in 2% acetonitrile (ACN) with 0.05% trifluoroacetic acid (TFA) and loaded onto a C18 trap column (AcclaimPepMap100 C18, Thermo Scientific; ID 75 μm, length 20 mm, particle size 3 μm, pore size 100 Å). Then, they were separated on a C18 analytical column (AcclaimPepMapRLSC C18, Thermo Scientific; ID 75 μm, length 500 mm, particle size 2 μm, pore size 100 Å) in a 4 h gradient of acetonitrile (2–40%) in the presence of 0.05% formic acid. Eluting peptides were ionized in a PicoView nanospray source (DPV-550, New Objective, Woburn, MA, USA) and measured with the mass spectrometer in a data-dependent mode using the Top12 method. The resolution of full MS and MS/MS scans was 70,000 and 17,500, respectively. The performance of the LC-MS/MS platform was monitored during data acquisition using the QCloud quality control system [53].

### 3.3. LC-MS/MS Data Processing

Data were processed using MaxQuant (version 1.6.7.0) [54] and searched by the integrated search engine, Andromeda [55], against Lacertidae SwissTrEMBL database (45,072 sequences, downloaded on 23 September 2020), supplemented with common protein contaminant sequences. iBAQ quantification was enabled and, except for this parameter, standard software settings were used, which included a false discovery rate (FDR) below 1% for peptide and protein identification. The resulting table with identified protein groups was further processed using Perseus [56]. Protein groups from the reverse database, common protein contaminants, as well as proteins only identified by site, were filtered out (with 1487 protein groups left). Data were log transformed and the matrix was filtered for protein groups that had valid iBAQ values in at least 10 out of 15 samples (667 protein groups). Means of the iBAQ values were calculated for each protein group to assess their abundance within femoral gland secretions. A heat map of the Pearson correlation coefficients, based on iBAQ values, was constructed with Perseus software to visually explore inter-individual variation in protein expression.

Protein groups were ranked according to their mean iBAQ values (from highest to lowest). Thirty proteins with the highest values (Top30) were manually grouped by function with the help of the information available in the UniProt database [34].

Functional enrichment analysis for the whole dataset (667 protein groups) was conducted using STRING [57,58]. Gene names were retrieved from the UniProt database for first major protein ID for each protein group. They were available for 586 proteins and all of them were uploaded to STRING. Human taxonomy was chosen to ensure a high degree of protein annotation. 510 of the genes were recognized by the software. Additionally, the DAVID bioinformatics resource [59,60] was applied, similarly using human taxonomy (506 recognized genes). The transfer of functional annotations between species is commonplace in proteomic and genomic studies [44,45,46,47]. Although the use of human reference databases (or other well-annotated databases) may increase the number of annotations, extrapolation of protein function to other organisms should be cautiously interpreted. Considering this limitation, we use a similar approach to provide tentative biological functions of FG proteins in sand lizards.

## 4. Conclusions

The use of mass spectrometry-based proteomics as a tool in chemical ecology may help identify and functionally annotate proteins found in the skin glands of vertebrates. We find that FG proteomes in sand lizards have a broad and complex composition, and reveal substantial variation in the total number of proteins among individuals. Nonetheless, some proteins were abundantly expressed across multiple individuals. At a functional level, FG proteins expressed at high intensity are related to the immunological response, lipid metabolism and antioxidant activity, among others, showing a striking similarity to protein groups identified in Galápagos marine iguanas. Although it is currently unknown whether the expression of immune-regulatory proteins occurs in FG secretions of other lizard species, our dataset demonstrates that these proteins are present in at least one other family of squamates (Lacertidae) beyond marine iguanas (family Iguanidae). Since marine iguanas are phylogenetically distant from lacertid lizards, it would be interesting to investigate whether other species secrete similar functional protein groups, and whether the expression of proteins related to immune response is a widespread phenomenon in squamate epidermal glands.

Proteins directly involved in intraspecific chemical communication were not identified in sand lizard FGs. However, some of the described proteins could have multiple functions, and thus further research is needed to validate the FG proteins found in sand lizards. Here, we provide a first assessment on the number, identity and tentative functions of FG proteins that should be explored in greater detail to understand other potential roles they may have in the context of intraspecific communication.

## Figures and Tables

**Table 1:** Thirty most abundant proteins (Top30) detected in the femoral gland secretions of sand lizards. Top30 proteins were manually grouped by function with the help of the information available in the UniProt database [34]. IR: Intensity Rank.

Protein ID	Protein Name	Gene Name	Length	Organism	IR *	IR in Tellkamp et al. **	Comment
Cathepsins							
D3JHM6_9SAUR	Cathepsin D (Fragment)	CTSD	24	*Lacerta schreiberi*	1	10	
A0A670IA46_PODMU	Peptidase A1 domain-containing protein	CTSD	399	*Podarcis muralis*	3	10	
A0A670IH97_PODMU	Carboxypeptidase (EC 3.4.16.-)	CTSA	530	*Podarcis muralis*	14	112	
Chaperones							
A0A670J0X0_PODMU	Uncharacterized protein	PDIA6	449	*Podarcis muralis*	4	not FG-specific ***	
A0A670KPF3_PODMU	78 kDa glucose-regulated protein (Binding-immunoglobulin protein) (Heat shock protein 70 family protein 5) (Heat shock protein family A member 5) (Immunoglobulin heavy chain-binding protein)	HSPA5	654	*Podarcis muralis*	5	17	
A0A670I8P5_PODMU	SHSP domain-containing protein		169	*Podarcis muralis*	8	not FG-specific ***	belongs to the small heat shock protein (HSP20) family
A0A670K8T4_PODMU	Uncharacterized protein	HSPA8	646	*Podarcis muralis*	11	not FG-specific ***	
Lipid related							
A0A670JAW3_PODMU	GP-PDE domain-containing protein	GDPD3	318	*Podarcis muralis*	6	not FG-specific ***	
A0A670JD81_PODMU	Beta-hexosaminidase (EC 3.2.1.52)	HEXA	522	*Podarcis muralis*	10	105	
A0A670HXK7_PODMU	Phospholipase A2 (EC 3.1.1.4)		731	*Podarcis muralis*	24	158	
A0A670KKI2_PODMU	Aldo_ket_red domain-containing protein		278	*Podarcis muralis*	28	not FG-specific ***	members of aldo-keto reductase family are involved in steroid metabolic processes
A0A670IG25_PODMU	Inositol-1-monophosphatase (EC 3.1.3.25)	IMPA1	347	*Podarcis muralis*	29	not FG-specific ***	
Cytoskeletal, Keratins							
A0A1D9CFN7_9SAUR	Beta-actin		375	*Eremias argus*	7	261	
A0A670KC37_PODMU	IF rod domain-containing protein	LOC114593000	611	*Podarcis muralis*	9	4, 7, 11, 12, 25, 46, 109, 764	keratin
A0A670JPK3_PODMU	IF rod domain-containing protein	LOC114581896	472	*Podarcis muralis*	27	4, 7, 11, 12, 25, 46, 109, 764	keratin
A0A670JN74_PODMU	IF rod domain-containing protein	LOC114582343	462	*Podarcis muralis*	30	4, 7, 11, 12, 25, 46, 109, 764	keratin
Histones							
A0A670K9E9_PODMU	Histone H2A	LOC114582712	129	*Podarcis muralis*	15	not FG-specific ***	
A0A670JD32_PODMU	Histone H3	LOC114581771	136	*Podarcis muralis*	16	not FG-specific ***	
A0A670K7U8_PODMU	Histone H4		101	*Podarcis muralis*	21	not FG-specific ***	
A0A670K3F4_PODMU	Histone H2B	LOC114581794	126	*Podarcis muralis*	22	not FG-specific ***	
Antioxidant defense							
A0A670I2N3_PODMU	Superoxide dismutase [Cu-Zn] (EC 1.15.1.1)	SOD1	155	*Podarcis muralis*	17	not FG-specific ***	
A0A670I4X7_PODMU	Thioredoxin domain-containing protein	PRDX4	276	*Podarcis muralis*	25	113	
Others							
A0A670JGA4_PODMU	Carbonic anhydrase (EC 4.2.1.1)	LOC114583714	316	*Podarcis muralis*	2	2	pH regulation
A0A670JY74_PODMU	Glyceraldehyde-3-phosphate dehydrogenase (EC 1.2.1.12)	GAPDH	333	*Podarcis muralis*	12	not FG-specific ***	
A0A670HW54_PODMU	40S ribosomal protein S27a (Ubiquitin carboxyl extension protein 80) (Ubiquitin-40S ribosomal protein S27a)	RPS27A	156	*Podarcis muralis*	13	204	
A0A670ICE5_PODMU	Elongation factor 1-alpha	EEF1A1	462	*Podarcis muralis*	18	398	
A0A670HUL4_PODMU	Voltage-dependent anion-selective channel protein 2	VDAC2	283	*Podarcis muralis*	19	not FG-specific ***	
A0A670HU92_PODMU	Mannosyl-glycoprotein endo-beta-*N*-acetylglucosaminidase (EC 3.2.1.96)	ENGASE	811	*Podarcis muralis*	20	94	glycoprotein modification
A0A670K0C8_PODMU	S_100 domain-containing protein	S100A14	102	*Podarcis muralis*	23	47	cell survival and apoptosis/epidermal identity
A0A670JM37_PODMU	SERPIN domain-containing protein	SERPINE2	386	*Podarcis muralis*	26	416	protease inhibitor

* iBAQ Intensity Rank—based on the mean value from at least 10 out of 15 samples. ** within femoral gland (FG) specific proteins in Galápagos marine iguanas [28]. *** identified in FG sample but not classified as FG-specific in Galápagos marine iguanas [28].

## Data Availability

The mass spectrometry data were deposited to the ProteomeXchange via the MassIVE repository with the dataset identifier PXD031566.

## Supplementary Materials

The following supporting information can be downloaded at: https://www.mdpi.com/article/10.3390/molecules27072371/s1, File S1. Information on the number of identified proteins, protein groups that had valid iBAQ values for at least 10 out of 15 samples, proteins retrieved from the UniProt database together with the intensity rank, STRING analysis and values for Figure 3A; File S2. Results of the analysis with the DAVID tool.

## Sample Availability

Samples of the compounds are not available from the author.
